# Influence of Teachers’ Grouping Strategies on Children’s Peer Social Experiences in Early Elementary Classrooms

**DOI:** 10.3389/fpsyg.2020.587170

**Published:** 2020-12-17

**Authors:** Saetbyul Kim, Tzu-Jung Lin, Jing Chen, Jessica Logan, Kelly M. Purtell, Laura M. Justice

**Affiliations:** ^1^Department of Educational Studies, The Ohio State University, Columbus, OH, United States; ^2^Graduate School of Education, Shanghai Jiao Tong University, Shanghai, China; ^3^Department of Human Sciences, The Ohio State University, Columbus, OH, United States

**Keywords:** teacher grouping strategies, friendship, peer conflict, early elementary classrooms, peer social experiences

## Abstract

Most children experience some form of grouping in the classroom every day. Understanding how teachers make grouping decisions and their impacts on children’s social development can shed light on effective teacher practices for promoting positive social dynamics in the classroom. This study examined the influence of teachers’ grouping strategies on changes in young children’s social experiences with peers across an academic year. A total of 1,463 children (51% girls, *M*_age_ = 6.79, *SD_age_* = 1.22) and 79 teachers from kindergarten to third-grade classrooms participated in this study. Teachers rated children’s behavioral problems as the most important consideration when creating seating charts or assigning children to small groups. Promoting existing or new friendships was rated as the least important consideration. Heterogeneous ability grouping, rated as somewhat important by the teachers, was associated with a decrease in children’s friendships and yet also a decrease in girls’ experience with peer conflicts. Our findings begin to fill in the gaps in the literature on the social impacts of ability grouping for young children.

## Introduction

The classroom is a primary social context in which school-age children experience various social interactions and relationships with peers. These peer social experiences have valence and can lead to long-term impacts on children’s social and academic development (Coplan and Arbeau, 2009; Oberle et al., 2010; Bulotsky-Shearer et al., 2012; Lin et al., 2016). As teachers are the key social agents with whom children spend the majority of their time in the classroom, they inevitably mediate children’s peer social experiences. This occurs in part through their daily instructional decisions or classroom management, such as determining classroom physical layout, governing with whom children collaborate, and maximizing cross-gender or cross-ethnic interactions through heterogeneous grouping (Gremmen et al., 2018). These teacher practices change the immediate social environment for children and their peers, which then shapes the social integration of the classrooms.

Despite the importance of teacher practices in children’s peer social experiences in classrooms (Gremmen et al., 2016), empirical evidence supporting the social influence of teachers’ practices remains scarce (Hallinan and Sørensen, 1985; Gest and Rodkin, 2011). Particularly, little attention has been paid to the social impacts of teachers’ grouping strategies, which refer to the ways by which teachers assign students in groups within classrooms for learning and instruction. This issue is important because children experience some forms of grouping by the teacher each day (Baines et al., 2003). These grouping practices mediate the physical proximity between dyads of children, which then alter their perception and interactions with one another (Van den Berg et al., 2012). To date, studies on teachers’ grouping strategies have primarily focused on how grouping affords teachers the opportunity to tailor instruction based on different children’s academic needs (see Saleh et al., 2007; Savanur et al., 2007; Nomi, 2009; Hong et al., 2012; Marks, 2014; Steenbergen-hu et al., 2016), with a few exceptions that examined the role of teachers’ grouping strategies in promoting more mixing or socially inclusive peer interactions and relationships (Gest and Rodkin, 2011; Van den Berg et al., 2012; McKeown et al., 2016).

To fill in this research gap, the purpose of this study was to examine the role of teachers’ grouping strategies in shaping children’s peer social experiences across the academic year in early elementary classrooms. Specifically, we focused on children’s friendship and peer conflicts because these social experiences emerge early in child development and together signify level of social inclusion in the classroom (Juvonen et al., 2019). Considering that boys and girls tend to show distinct profiles of socially competent behavior (Underwood, 2007; Card et al., 2008; Godinet et al., 2014; Shin, 2017), we further examined whether teachers’ grouping strategies have differential impact on boys’ and girls’ friendship and conflict experiences.

## Literature Review

### Peer Social Experiences in Early Childhood Classrooms

Children begin to form positive and negative experiences with their classroom peers as young as preschool age (Ladd and Price, 1987; Howes, 1988; Ladd, 1990). These experiences with peers have shown long-term influences on children’s social and academic development (Boulton and Smith, 1994; Coplan and Arbeau, 2009; Oberle et al., 2010; Bulotsky-Shearer et al., 2012; Lin et al., 2016). Positive experiences such as friendships can provide a context for cooperation and negotiation (Carter and Nutbrown, 2016) and ease children’s adjustment to school life (Margetts, 2002; Corsaro, 2003; Peters, 2003). Meanwhile, negative peer experiences such as conflict or aggression can hinder children’s self-worth, social competence, and school engagement (Kamper-DeMarco and Ostrov, 2019), leading to loneliness, depression, and school dropout (Buhs et al., 2006; Meyer and Ostrosky, 2018).

While classroom peer experiences can involve various relational aspects, in this study we focus on children’s friendship and peer conflicts, both of which are the most prevalent peer experiences in young children, and can lead to a wide range of socioemotional and academic difficulties across the life span, such as school failure and dropout (Coie and Dodge, 1998; Chang, 2003; Kutnick and Kington, 2005; Shin, 2017; Kamper-DeMarco and Ostrov, 2019). Research shows that as young as preschoolers, at least 83 percent of children in the classroom were engaged in friendships (Quinn and Hennessy, 2010), and the number of friends that a child makes increases as they transition to first grade (Hartup, 1992). Friendships are ‘egalitarian in nature’ (Schaffer, 1996, p.312), providing a relational context for children to practice social integration with others (e.g., conflict resolution, empathy, negotiation, Cillessen and Marks, 2017). In this aspect, friendship relationships are key to promoting an inclusive and supportive classroom atmosphere (Division for Early Childhood, and National Association for the Education for Young Children, 2009).

Peer conflicts, often revealed in the form of physical aggression or verbal dispute in young children, is normative and tend to occur at high rates in the classroom (Odgers et al., 2008). It occurs when children have incompatible needs, wishes, or goals with one another (Hay, 1984). In a study based on the Early Childhood Longitudinal Study, Kindergarten Class of 1988-99 (ECLS-K), at least 10% of children in kindergarten experienced frequent arguments and fights with peers (West et al., 2001). One in every four to six children (15–23%) are victims of aggression in primary school settings (Robers et al., 2012). It is crucial for children to experience peer conflict as it provides children opportunities to practice perspective taking, conflict mitigation, and social-emotional regulation (Eisenberg and Garvey, 1981; Rende and Killen, 1992; Malloy and McMurray, 1996; Miller et al., 2004). However, escalated conflicts in the classroom can cause negative emotion and stress, damage social relationships, and hinder children’s school adjustment (Blair, 2002).

Together, friendships and peer conflicts comprise children’s important social experiences that can have significant impacts on their social, emotional, and academic development from the early phase of lives through later developmental stages (Bulotsky-Shearer et al., 2012; Kamper-DeMarco and Ostrov, 2019). It is critical to identify key contextual factors that would hinder or promote children’s peer social experiences. By this, we examined teachers’ grouping strategies.

### Teachers’ Grouping Strategies and Children’s Peer Social Experiences

Putting children in groups is one of the everyday teacher practices in the classroom. Grouping can take place in various forms and structures (e.g., small groups, dyads, and classroom seating positions) and varies by teachers’ purposes and strategies. For instance, teachers may assign children to work with their same-ability peers with the goal of tailoring instruction based on children’s different academic needs (Patrick, 2020). Teachers may form groups of children with diverse skills with the aim at stimulating diverse thinking (Murphy et al., 2017) or promoting social inclusion and equity (Cohen et al., 1999). When making a seating chart, teachers may take into account children’s existing peer relationships or social behavior with the goals of maintaining classroom order and social cohesion (Gest and Rodkin, 2011). It stands to reason that teachers’ attitudes toward grouping strategies may reflect their instructional priority and beliefs about peer influence in the classroom.

Among various grouping strategies, ability grouping is the most common and yet controversial grouping strategy (Slavin, 1987; Hallam and Parsons, 2013). One reason is that children’s academic achievement is often a correlate of social status in early childhood years (Rubin et al., 2006). Grouping children by ability levels may either increase or flatten the social hierarchy in the classroom, which then shape their social experiences with peers. To date, however, ability grouping has mostly been associated with students’ academic performance (Sørensen and Hallinan, 1986; Slavin, 1987; Dreeben and Barr, 1988; Wilkinson, 1989). Research that examined the effects of ability grouping on children’s social experiences is relatively scarce, most of which focused on children’s self-esteem, self-concepts if not academic attainment (e.g., Oakes, 1985; Gamoran and Berends, 1987; Kulik and Kulik, 1992; Ireson et al., 2001; Suk Wai Wong and Watkins, 2001; Ireson and Hallam, 2005, 2009).

There are two contrasting ability grouping strategies. *Homogeneous ability grouping* is known for its positive impacts on children’s achievement (MacIntyre and Ireson, 2002). In addition, working with like-minded classmates may increase children’s sense of belonging (Riley and White, 2016) and support teachers’ instructional differentiation (Patrick, 2020). It is criticized, however, for its potential harmful effects on the self-concepts and well-being of children with lower abilities (Marsh, 1984; Oakes, 1985). *Heterogeneous ability grouping* is assumed to enhance learning and interdependence because working with peers with diverse skills may allow children to recognize gaps in their own thinking and to foster a sense-making process when more competent children provide explanations and support to less competent peers (Wilkinson et al., 2010). However, there is always a concern about sacrificing high-ability children’s learning opportunity in heterogeneous ability grouping (Mashburn et al., 2009).

Other teacher grouping strategies consider children’s existing or potential peer relationships based on the assumption that sitting or working with classmates in close proximity allows children to learn about each other better, which then change their relationships with one another (Pettigrew, 1998). Research on seating assignment demonstrates that by manipulating children’s seating positions, children who did not like each other at the beginning of the school year showed higher likability with each other as a result of close proximity (Van den Berg et al., 2012). This influence of near-seated peers has been examined in both classroom and small groups settings (Webb, 1989; Barth et al., 2004; Burke and Sass, 2013; Gremmen et al., 2018). The findings suggest that physical proximity likely increases the likelihood of interaction between children; the increased interaction help children recognize their similarity and develop positive sentiments to each other (Homan, 1974). On the contrary, the absence of proximity may create barriers for friendship formation (Hallinan and Tuma, 1978).

Another common grouping strategy draws attention to children’s behavioral problems. Children’s behavioral problems have been noted as one of the prominent factors that disrupt classroom learning and instruction in early childhood classrooms (Rimm-Kaufman et al., 2000; Gutman et al., 2003). Findings have been mixed regarding whether managing children’s behavioral problems (e.g., fights, quarrel, and aggression) can have a positive influence on children’s social development (Singer and Hännikäinen, 2002; Spivak, 2016). For instance, Gest and Rodkin (2011) showed that teachers who placed strong emphasis toward separating children with behavioral problems had students who expressed a stronger liking to each other and reported denser friendship networks. Other research showed that intervening in peer conflicts by directly separating the conflict children as opposed to helping children develop mutually agreed upon solutions can lead to negative conflict outcomes (Myrtil et al., unpublished).

Taken together, the existing literature suggests that teachers make grouping strategies based upon various factors, including children’s ability level, peer relationships, and problem behaviors. Yet, findings are far from conclusive regarding how these grouping strategies impact children’s social experiences with peers. The current study aimed to address this research gap.

### Gender Effects in Peer Social Experiences

Ample theories and research highlight the importance of gender in children’s peer social experiences. A substantial body of research has shown that boys tend to show more externalizing behaviors (e.g., physical aggression) and have more frequent conflicts with peers than girls (Hamre and Pianta, 2001; Saft and Pianta, 2001; Crick et al., 2006; Graves and Howes, 2011). On the contrary, girls have been found to demonstrate more prosocial behaviors and intimate friendships than boys (Berndt and Perry, 1986; Chung and Asher, 1996; Rose and Asher, 2004; Van Leeuwen et al., 2006). Other studies show that boys value independence and social dominance while girls place more emphasis on harmonious relationships (see Rose and Rudolph, 2006).

Given the gender differences in peer interactions and relationships, teachers’ grouping strategies may have differential effects on boys’ and girls’ peer social experiences in the classroom. It is likely that teachers may knowingly or unknowingly treat boys and girls differently when applying the same grouping strategy in the classroom (Troop-Gordon and Ladd, 2015). For instance, teachers may separate more boys than girls because boys’ conflicts or aggressive behaviors are more frequent and salient than girls’ conflicts. Research shows that the average rate at which teacher react to children’s aggressive behavior was over three times higher for boys compared to girls (Serbin et al., 1973). Alternatively, boys and girls may react to teachers’ grouping strategies differently, leading to different social experiences with peers. For instance, when working with classmates in heterogeneous groups, boys may be less coordinated, more impulsive, and show more disruptive behaviors than girls (Serbin et al., 1973) and therefore benefit less from working with peers with diverse skills.

### The Current Study

This study was part of a large-scale, federally funded project designed to understand the classroom ecology in preschool to third-grade classrooms. The data were collected from two large, suburban school districts in a midwestern city in the United States, including 2090 students from 96 classrooms in 33 schools. The two school districts are representative of the socio-economic and racial diversity of adjacent suburban communities. This study excluded the preschool sample based on the consideration that teachers’ grouping practices in preschool classrooms might be fundamentally different from those in early elementary classrooms due to more focuses on free play and non-academic learning (Justice et al., 2020). In addition, preschoolers might not have developed the same level of ability to reliably report their social experiences compared to other older children in this study (Chen et al., 2020).

Children’s peer social experiences in the classroom was assessed using a sociometric method in which children were asked to nominate an unlimited number of classmates who fit certain selection criteria. Peers are key informants of children’s social experiences because they spend a significant amount of time with children under various social situations (Rubin and Cohen, 1986; Coie and Dodge, 1988). Gathering classroom peers’ perceptions of a child’s social experiences provides higher level of objectivity than the self-report or parent report (Clark and Ladd, 2000). We employed the unlimited nomination approach because research suggests that unlimited nominations can capture children’s social relationships more comprehensively and reliably than the limited nominations approach (Cillessen and Mayeux, 2004; Cillessen and Borch, 2006; Cillessen and Marks, 2017).

Teachers’ grouping strategies were assessed based on the teacher-reported measure developed by Gest and Rodkin (2011). In their study, first to fifth grade teachers were asked to rate the importance of five different grouping strategies when they created a seating chart or assigned children in groups: (a) reinforcing existing friendships, (b) promoting new friendships, (c) ability grouping with homogeneous skill levels, (d) ability grouping with diverse skill levels, and (e) separating students with behavioral problems. They found that teachers generally considered separating students with behavioral problems as the most important grouping strategy, followed by promoting academic diversity and new friendships. Teachers did not place a strong consideration on forming academic homogeneity or reinforcing existing friendships. Furthermore, classrooms tended to have a higher ratio of liking to disliking and a higher density of friendships if the teachers reported that separating students with behavior problems was a major consideration in creating seating charts and small groups. In this study, we considered teacher-reported grouping strategies as a proxy of teachers’ actual grouping practices because previous research suggests that teachers’ attitudes and beliefs drive their instructional decisions (Fang, 1996; Vartuli, 1999; Muijs and Reynolds, 2002; Missett et al., 2014).

In all, three research questions are addressed in this study: (1) How do teachers from kindergarten to third grade incorporate grouping strategies in their daily instruction? Based on the pioneering study conducted by Gest and Rodkin (2011), we hypothesize that early elementary teachers might consider separating students with behavioral problems the most important grouping strategy for creating a seat chart of forming students in groups. Teachers may pay the least attention to reinforcing existing friendships. (2) Are teachers’ grouping strategies associated with changes in children’s peer social experiences across the academic year? We hypothesize that grouping strategies that are rated as more important by the teachers would be more associated with changes in children’s peer social experiences. (3) Are the associations between teachers’ grouping strategies and changes in children’s peer experiences moderated by gender? Based on the literature, we hypothesize that gender can have a significant moderation effect on the association between teachers’ grouping strategies and children’s peer experiences.

To address these research questions, we controlled for children’s gender, disability status, dual language status, and maternal education based on previous findings suggesting that friendships and peer conflicts can vary by these demographic characteristics. Research shows that girls are more likely to have best friends than boys (Sebanc et al., 2007). Boys tend to exhibit more physical aggression (Crick et al., 2006; Juliano et al., 2006) while girls are more relationally aggressive than boys (Crick et al., 2004; Ostrov et al., 2004). Older children tend to have more friends than younger children (Sebanc et al., 2007). Children with lower socioeconomic status (Bradley et al., 2001; Raver and Knitzer, 2002), different linguistic backgrounds (Eslea and Mukhtar, 2000), and disabilities (Hemmeter et al., 2006; Odom et al., 2006) are more at risk for negative peer social experiences. Finally, we controlled for teachers’ years of teaching and self-efficacy for managing peer relationships (e.g., How much can you help students make friends at school?) in the classroom because both have been found to associate with classroom quality (Swanson et al., 1990; Brophy, 2006; Watson, 2006; Nahal, 2010; Gebbie et al., 2012; Ryan et al., 2015).

## Materials and Methods

### Participants

The sample includes 1,463 children and 79 teachers from 20 public elementary schools located in two suburban districts in a midwestern city. This was after removing the preschool sample and one kindergarten teacher and her students because the teacher did not fill out the teacher survey. Children [girls = 51% (Kindergarten: 42.9%, Grade 1: 18.1%, Grade 2: 22.3%, Grade 3: 16.7%)] with an average age of 6.79 years (*SD* = 1.22). About 14.8% of children were dual language learners and a total of 7.8% were in individualized education plan (IEP). Many children were White (61.1%). The distribution of other race and ethnicity categories were Black (4.5%), Asian (8.5%), Multi-racial (6.2%), and Other (2.0%). Teachers were mostly female (98.7%) and White (92.4%). About 73.4% of teachers had a master’s degree, followed by 19.0% with bachelor’s degree, 2.5% with other degrees, and 5.1% who did not report their education level. Years of teaching experience ranged from 2 to 35 years (mean = 14.21).

### Measure

#### Peer Social Experiences

The peer nomination approach (Parkhurst and Asher, 1992; Chen et al., 2020) was used to assess children’s peer social experiences. In the fall and spring, children received individual interviews with field assessors to nominate unlimited number of children in class who fit the nomination descriptions. Children were given a class roster containing pictures of classmates in order to reduce the cognitive need to recall names for nominations. Two items were used in this study to assess three aspects of peer social experiences: (a) conflicts: “In your classroom, who gets into fights with other kids?,” and (b) friendship: “In your classroom, who are your best friends?” Previous studies show that using single peer nomination item to assess a unique aspect of social experiences can yield satisfactory psychometric property (van den Berg and Cillessen, 2013; Babcock et al., 2014). Even for children as young as preschoolers, their peer nominations yield congruent representations of peer social experiences with teachers’ reports and researchers’ observations (Chen et al., 2020). The number of nominations each child received from their classmates was calculated and used to indicate the degree with which each child experienced peer conflicts and developed friendships in their classroom. In the current study, number of nominations children received in the fall was significantly correlated with those in the spring (*r*s = 0.56 and 0.51, *p*s < 0.01 for peer conflicts and friendships, respectively).

#### Teachers’ Grouping Strategies

Adapted from Gest and Rodkin’s (2011) scale, teachers reported the extent to which five grouping strategies were important as they created the seating chart and assigning students to a small group: (a) reinforcing existing friendships, (b) promoting new friendships, (c) ability grouping with homogeneous skill levels, (d) ability grouping with diverse skill levels, and (e) separating students with behavioral problems. Teachers reported their grouping strategies based on a 3-point Likert scale (0 = not at all important, 1 = somewhat important, 2 = very important). The ratings under two different settings (creating a seat chart, small grouping) were average for each grouping strategy.

### Procedure

Teachers completed surveys about their instructional practices and beliefs, perceptions of children in the classrooms and demographic information online *via* the Qualtrics platform or on paper (based on their preference) during the spring semester of the school year. Paper surveys were converted to digital forms via a Teleform system. Trained research staff conducted quality assurance checks of scanned data, conducting a mandatory visual check of each scanned form for accuracy. In addition, data were checked to ensure data were all within the potential observable range for each variable, examined data for consistency between item and sum or total scores. Children’s classroom peer experience was collected by trained project staff in the fall and spring of the year. Children were interviewed in quiet areas of the hallway by trained research staff and responses were recorded in accordance with the study protocols.

### Data Analysis

To examine whether teachers’ grouping strategies were associated with changes in children’s peer experience in the classroom, hierarchical generalized linear models were performed in which each type of peer social experiences was the dependent variable predicted by teachers’ grouping strategies. Peer nominations of friendships and conflicts followed the Poisson distribution. As children were nested within classrooms (Level 1: child; Level 2: class), a random effect of intercept was specified in each model. To examine the gender moderation effect, the interaction of gender with each grouping strategy was examined.

#### Missing Data

Due to the non-negligible proportion of missing values (∼25%) in participants’ demographic information (i.e., IEP, DLL), additional testing was performed to determine if data were missing completely at random (MCAR) using Little’s MCAR test. Aside from IEP and DLL, percentage of missing ranged from 0.2% (gender) to 17.8% (ethnicity). The IEP and DLL variables were missing at 23 and 26%, respectively. Based on Little’s MCAR test, the pattern of missingness was not completely at random and therefore, listwise deletion would not be appropriate (Graham, 2012). We performed multilevel multiple imputation using a fully conditional specification (FCS) imputation approach in Blimp (Enders et al., 2018). Variables included in the multiple imputations were all the study variables as well as auxiliary variables that were related to missingness (Schafer and Olsen, 1998). Twenty imputed datasets were generated and analyzed using Proc Glimmix in SAS. Proc Mianalyze was used to combine statistical results and generate valid statistical inferences about each parameter.

## Results

### Exploratory Analyses

Table 1 presents the child-level descriptive statistics of the variables used in the current study. On average, children received 1.01 nomination from classmates for engaging in peer conflicts at the beginning of the academic year. The number of nominations increased to 1.64 at the end of the year. Children’s friendship nomination was 2.93 on average at the beginning of the year and decreased to 2.79 at the end of the year. Paired *t*-tests based on the imputed data set showed that children were perceived by peers as being more aggressive in the spring compared to that in the fall (*t* = 3.11, *p* < 0.01). Meanwhile, children received fewer friendship nominations in the spring than in the fall (*t* = −2.73, *p* < 0.01).

**TABLE 1:** Child-related descriptive analysis.

	**% Missing**	**%**	**Min**	**Max**	**Mean**	**SD**
Gender (0 = Boys, 1 = Girls)	0.2	51.1	0.0	1.0		
Age in years	0.3		4.3	9.5	6.79	1.22
Ethnicity	17.8					
White		61.4				
Black		4.5				
Asian		8.5				
Other		2.0				
Multi-racial		6.2				
Grade	0.0		0.0	4.0		
Kindergarten		42.9				
Grade 1		18.1				
Grade 2		22.3				
Grade 3		16.7				
IEP (0 = No, 1 = Yes)	23.0	10.1	0.0	1.0		
DLL (0 = No, 1 = Yes)	25.8	20.0	0.0	1.0		
Maternal Education	17.6		0.0	4.0		
<high school		3.3				
high school		17.8				
associate		9.4				
bachelor’s		28.2				
Graduate or professional		23.6				
Peer social experiences						
Peer conflicts (fall)	1.6		0.0	13	1.01	1.80
Friendship (fall)	1.6		0.0	13	2.96	2.03
Peer conflicts (spring)	0.0		0.0	14	1.16	2.06
Friendship (spring)	0.0		0.0	11	2.79	2.02

*IEP, individualized education plan; DLL, dual language learner.*

Analysis of Variance (ANOVA) tests were conducted to explore if the patterns of change differed by children’s gender. The conflict nominations received by children at the beginning of the year were 1.49 for boys, and 0.56 for girls, and this difference was statistically significant [*t* = 10.10, *p* < 0.001]. At the end of the academic year, boys continued to receive more physical aggression nominations than girls [*M_boy_* = 1.65, *M*_girl_ = 0.70; *t* = 9.07, *p* < 0.001]. Children’s friendship showed the opposite trend. Girls received more friendship nominations than boys in the fall [*M_boy_* = 2.83, *M*_girl_ = 3.04; *t* = −1.98, *p* < 0.05], but this difference was not statistically significant in the spring [*M_boy_* = 2.71, *M*_girl_ = 2.87; *t* = −1.49, *p* < 0.14].

Among the 79 teachers, 89.9% answered ‘yes’ to a survey question about whether they created a seating chart in the classrooms (the other 10.1% did not respond to this question); 88.6% teachers answered ‘yes’ to a survey question about whether they let students work in small groups (the other 11.4% did not respond to this question).

### Teacher-Reported Importance of Grouping Strategies

Table 2 shows the descriptive statistics and the correlations between teachers’ grouping strategies based on teachers’ reports. In response to the first research question, teachers rated separating behavioral problems as most important (*M* = 1.91 out of the maximum value of 2.00), followed by heterogeneous ability grouping (*M* = 1.42) and homogeneous grouping (*M* = 1.20). On average, teachers regarded reinforcing existing friendships the least important (*M* = 0.61). Promoting new friendships was rated slightly higher than reinforcing existing friendships (*M* = 1.17).

**TABLE 2:** Descriptive of teachers’ grouping strategies.

	***Mean***	***SD***	**1.**	**2.**	**3.**	**4.**
1. Existing Friendship	0.61	0.52				
2. New Friendship	1.17	0.50	0.35**			
3. Homogeneous Ability Grouping	1.20	0.49	0.34**	0.24*		
4. Heterogeneous Ability Grouping	1.42	0.41	0.23*	0.17	0.31**	
5. Behavioral Problems	1.91	0.26	0.07	0.09	0.14	0.06

**p < 0.01, *p < 0.05.

Reinforcing existing friendship was moderately correlated with promoting new friendships (*r* = 0.35), homogeneously ability grouping (*r* = 0.34), and heterogeneous ability grouping (*r* = 0.23). Promoting new friendships was moderately correlated with homogeneous ability grouping (*r* = 0.24). Homogeneous ability grouping was moderately correlated with heterogeneous ability grouping (*r* = 0.31). Teacher rating of separating students with behavioral problems was not significantly correlated with any other grouping strategies, which indicates that this grouping strategy is distinct from any other grouping strategies. Overall, all of the correlations were positive, suggesting that teachers who perceived one grouping strategy as important were likely to consider another grouping strategy as important as they created seating charts or assigned groups.

### Teachers’ Grouping Strategies and Children’s Peer Social Experiences

Table 3 presents fixed effects of teachers’ grouping strategies on children’s conflicts based on the imputed data. None of the grouping strategies significantly predicted changes in children’s conflicts over the academic year, after controlling for children’s demographic characteristics, years of teaching, and teachers’ self-efficacy for managing peer relationships. Gender was found to significantly predict children’s conflicts: Girls had lower levels of conflicts than boys [*b* = −0.56, *exp*(b) = 0.57, *SE* = 0.06, *p* < 0.001]. Higher teacher self-efficacy for managing children’s peer relationships was associated lower peer conflicts [*b* = −0.21, *exp*(b) = 0.81, *SE* = 0.10, *p* < 0.05].

**TABLE 3:** Predicting changes in peer conflicts by teachers’ grouping strategies.

	**Peer Conflicts**				
	***b***	***Exp(b)***	***SE***	***t***	***95% CI***
Intercept	1.66***	5.26	0.45	3.65	[0.77, 2.55]
Gender (0 = Boys, 1 = Girls)	−0.56***	0.57	0.06	−10.07	[−0.67, −0.45]
IEP (0 = No, 1 = Yes)	0.01	1.01	0.11	0.12	[−0.20, 0.23]
DLL (0 = No, 1 = Yes)	−0.10	0.90	0.12	−0.82	[−0.33, 0.14]
Grade 1	0.20	1.22	0.12	1.63	[−0.04, 0.43]
Grade 2	0.14	1.15	0.12	1.19	[−0.09, 0.37]
Grade 3	−0.04	0.96	0.13	−0.29	[−0.29, 0.21]
Maternal Education	−0.04	0.96	0.03	−1.13	[−0.11, 0.03]
Peer conflict pre-test (Fall)	0.14***	1.15	0.01	21.8	[0.12, 0.15]
Teacher experience	−0.00	1.00	0.01	−0.52	[−0.01, 0.01]
Teacher efficacy	−0.21*	0.81	0.10	−2.17	[−0.40, −0.02]
**Grouping Strategies**					
Existing Friendship	−0.03	0.97	0.08	−0.38	[−0.19, 0.12]
New Friendship	0.05	0.95	0.08	0.62	[−0.11, 0.21]
Homogeneous Ability	0.01	1.01	0.09	0.11	[−0.17, 0.19]
Heterogeneous Ability	−0.03	0.97	0.11	−0.30	[−0.25, 0.18]
Behavioral Problem	−0.05	0.95	0.14	−0.34	[−0.33, 0.23]

The reference group of Grade was Kindergarten; IEP, individualized education plan; DLL, dual language learner; Maternal education (1 = Higher than an associate degree; 0 = otherwise). ***p < 0.001, **p < 0.01, *p < 0.05.

Table 4 demonstrates the fixed effects of teachers’ grouping strategies on children’s friendships. After controlling for the covariates, heterogeneous ability grouping negatively predicted children’s friendships [*b* = −0.14, *exp*(b) = 0.87, *SE* = 0.07, *p* < 0.05]. Keeping everything else constant, with one unit of increase in teacher-reported importance of heterogeneous ability grouping, children’s friendship nominations would decrease by 13%. Children who were in IEP showed lower levels of friendships than typically developing children [*b* = −0.23, *exp*(b) = 0.79, *SE* = 0.08, *p* < 0.01].

**TABLE 4:** Predicting changes in friendships by teachers’ grouping strategies.

	**Friendships**				
	***b***	***Exp(b)***	***SE***	***t***	***95% CI***
Intercept	1.31***	3.71	0.31	4.22	[0.70, 1.93]
Gender (0 = Boys, 1 = Girls)	0.02	1.02	0.03	0.47	[−0.05, 0.08]
IEP (0 = No, 1 = Yes)	−0.23**	0.79	0.08	−2.75	[−0.40, −0.06]
DLL (0 = No, 1 = Yes)	−0.09	0.91	0.06	−1.59	[−0.20, 0.02]
Grade 1	0.03	1.03	0.08	0.39	[−0.12, 0.18]
Grade 2	0.02	1.02	0.07	0.31	[−0.12, 0.17]
Grade 3	−0.12	0.89	0.08	−1.46	[−0.27, 0.04]
Maternal Education	0.02	1.02	0.02	0.84	[−0.02, 0.06]
Friendship pre-test (Fall)	0.13***	1.14	0.01	13.91	[0.11, 0.15]
Teacher experience	0.00	1.00	0.00	1.06	[−0.00, 0.01]
Teacher efficacy	−0.10	0.90	0.06	−1.56	[−0.23, 0.03]
**Grouping Strategies**					
Existing Friendship	0.02	1.02	0.05	0.30	[−0.09, 0.12]
New Friendship	0.06	1.06	0.05	1.07	[−0.05, 0.16]
Homogeneous Ability	0.03	1.03	0.06	0.59	[−0.08, 0.15]
Heterogeneous Ability	−0.14*	0.87	0.07	−1.98	[−0.27, −0.00]
Behavioral Problem	−0.01	0.99	0.09	−0.06	[−0.19, 0.18]

The reference group of Grade was Kindergarten; IEP, individualized education plan; DLL, dual language learner; Maternal education (1 = Higher than an associate degree; 0 = otherwise). ***p < 0.001, **p < 0.01, *p < 0.05.

### Gender Effects in the Relationship Between Teachers’ Grouping Strategies and Peer Social Experiences

As shown in Table 5, children’s gender was found to interact with heterogeneous ability grouping in predicting children’s conflicts [*b* = −0.30, *exp*(b) = 0.74, *SE* = 0.15, *p* < 0.05]. Specifically, heterogeneous ability grouping strategies negatively lowered girls’ conflicts but not boys’. The effect of teacher-efficacy for managing children’s peer relationships remained significant [*b* = −0.21, *exp*(b) = 0.81, *SE* = 0.10, *p* < 0.05].

**TABLE 5:** Interactive effects of gender and teachers’ grouping strategies on changes in peer conflicts across the academic year.

	**Peer Conflicts**				
	***b***	***Exp(b)***	***SE***	***t***	***95% CI***
Intercept	1.66***	5.26	0.48	3.47	[0.72, 2.59]
Gender (0 = Boys, 1 = Girls)	−0.55	0.58	0.43	−1.29	[−0.14, 0.28]
IEP (0 = No, 1 = Yes)	0.02	1.02	0.11	0.20	[−0.19, 0.24]
DLL (0 = No, 1 = Yes)	−0.10	0.90	0.12	−0.86	[−0.34, 0.14]
Grade 1	0.20	1.22	0.12	1.64	[−0.04, 0.44]
Grade 2	0.14	1.15	0.12	1.15	[−0.10, 0.37]
Grade 3	−0.04	0.96	0.13	−0.28	[−0.29, 0.22]
Maternal Education	−0.04	0.96	0.04	−1.07	[−0.11, 0.03]
Peer conflict pre-test (Fall)	0.14***	1.15	0.01	21.76	[0.12, 0.15]
Teacher experience	−0.00	1.00	0.01	−0.51	[−0.01, 0.01]
Teacher efficacy	−0.21*	0.81	0.10	−2.19	[−0.41, −0.02]
**Grouping Strategies**					
Existing Friendship	−0.07	0.93	0.09	−0.79	[−0.24, 0.10]
New Friendship	0.11	1.12	0.09	1.23	[−0.07, 0.28]
Homogeneous Ability	−0.02	0.98	0.10	−0.19	[−0.22, 0.18]
Heterogeneous Ability	0.06	1.06	0.12	0.52	[−0.17, 0.29]
Behavioral Problem	−0.12	0.89	0.16	−0.75	[−0.43, 0.19]
**Gender × Grouping Strategies**					
Gender × Existing Friendship	0.13	1.14	0.11	1.14	[−0.09, 0.35]
Gender × New Friendship	−0.21	0.81	0.11	−1.87	[−0.43, 0.01]
Gender × Homogeneous Ability	0.09	1.09	0.12	0.73	[−0.15, 0.33]
Gender × Heterogeneous Ability	−0.30*	0.74	0.15	−2.06	[−0.59, −0.01]
Gender × Behavioral Problem	0.25	1.28	0.21	1.20	[−0.16, 0.65]

The reference group of Grade was Kindergarten; IEP, individualized education plan; DLL, dual language learner; Maternal education (1 = Higher than an associate degree; 0 = otherwise). ***p < 0.001, **p < 0.01, *p < 0.05.

Table 6 shows a negative main effect of heterogeneous ability grouping on changes in children’s friendships [*b* = −0.21, *exp*(b) = 0.81, *SE* = 0.08, *p* < 0.05]. None of the other interaction effects was significant. The effect of IEP remained significant [*b* = −0.23, *exp*(b) = 0.79, *SE* = 0.08, *p* < 0.01].

**TABLE 6:** Interactive effects of gender and teachers’ grouping strategies on changes in friendships across the academic year.

	**Friendships**				
	***b***	***Exp(b)***	***SE***	***t***	***95% CI***
Intercept	1.38***	3.97	0.34	4.04	[0.71, 2.05]
Gender (0 = Boys, 1 = Girls)	−0.14	0.87	0.25	−0.58	[−0.63, 0.34]
IEP (0 = No, 1 = Yes)	−0.23**	0.79	0.08	−2.75	[−0.40, −0.06]
DLL (0 = No, 1 = Yes)	−0.09	0.91	0.06	−1.59	[−0.20, 0.02]
Grade 1	0.03	1.03	0.08	0.37	[−0.12, 0.18]
Grade 2	0.02	1.02	0.07	0.31	[−0.12, 0.17]
Grade 3	−0.12	0.89	0.08	−1.48	[−0.28, 0.04]
Maternal Education	0.02	1.02	0.02	0.90	[−0.02, 0.06]
Friendship pre-test (Fall)	0.13***	1.14	0.01	13.95	[0.11, 0.15]
Teacher experience	0.00	1.00	0.00	1.07	[−0.00, 0.01]
Teacher efficacy	−0.10	0.90	0.06	−1.51	[−0.22, 0.03]
**Grouping Strategies**					
Existing Friendship	0.02	1.02	0.06	0.32	[−0.11, 0.15]
New Friendship	0.07	1.07	0.06	1.14	[−0.05, 0.20]
Homogeneous Ability	0.10	1.11	0.07	1.46	[−0.03, 0.24]
Heterogeneous Ability	−0.21*	0.81	0.08	−2.48	[−0.37, −0.04]
Behavioral Problem	−0.05	0.95	0.12	−0.44	[−0.28, 0.18]
**Gender × Grouping Strategies**					
Gender × Existing Friendship	−0.01	0.99	0.07	−0.12	[−0.15, 0.13]
Gender × New Friendship	−0.03	0.97	0.06	−0.54	[−0.16, 0.09]
Gender × Homogeneous Ability	−0.13	0.88	0.07	−1.74	[−0.27, 0.02]
Gender × Heterogeneous Ability	0.13	1.14	0.08	1.54	[−0.04, 0.30]
Gender × Behavioral Problem	0.09	1.09	0.12	0.79	[−0.14, 0.33]

The reference group of Grade was Kindergarten; IEP, individualized education plan; DLL, dual language learner; Maternal education (1 = Higher than an associate degree; 0 = otherwise). ***p < 0.001, **p < 0.01, *p < 0.05.

## Discussion

This study sought to deepen our understanding of teachers’ grouping strategies and their roles in children’s peer social experiences in early elementary classrooms. Based on classroom peers’ observations, children in this study experienced a decreasing trend of friendship development and an increasing rate of peer conflicts across the academic year. Changes in these peer social experiences were predicted by teacher-reported importance of heterogeneous ability grouping. Specifically, children experienced greater loss in friendships in the classroom if their teachers viewed heterogeneous ability grouping as an important grouping strategy. Contrary to its negative influence on friendship development, teacher-reported importance of heterogeneous ability grouping was found to alleviate girls’ but not boys’ peer conflicts. Overall, our findings partially support the hypothesis that teachers can mediate children’s peer social experiences through various grouping strategies. The social impacts of grouping strategies seem to operate in more indirect and implicit ways.

Consistent with Gest and Rodkin’s (2011) findings, teachers in this study reported viewing strategies for separating students with behavior problems as more important than ability grouping or strategies for forming existing or new friendships strategies when they create seating charts or form small groups. This finding is also aligned with the conflict intervention literature showing that early childhood teachers tend to intervene in peer conflicts mainly when the conflicts escalate (Myrtil et al., unpublished); when the teachers intervene, they tend to use more cessation strategies (e.g., directly separating conflict peers) than mediation strategies (e.g., guiding students to resolve conflicts via negotiation, Spivak, 2016). Contrary to the positive association between separating behavioral problems and peer liking documented in Gest and Rodkin’s study with first, third, and fifth grade students, separating behavioral problems did not predict changes in children’s friendships or peer conflicts in our study. This seems to suggest that early elementary teachers tend to base their grouping decisions on children’s overt, salient characteristics. Teachers’ attunement to children’s behavioral problems may be at the expense of other factors might be more directly linked to children’s peer social experiences. The non-significant associations between the separating behavioral problems strategy and the other grouping strategies support this explanation.

Another major finding of this study is the negative influence of teacher-reported heterogeneous ability grouping on children’s friendship development. A rich body of social network research has documented that children tend to befriends peers with whom they share similar characteristics, such as gender, age, or ability levels, called the homophily phenomenon (Brechwald and Prinstein, 2011; Hafen et al., 2011; Ojanen et al., 2013). By assigning children of diverse ability into the same groups, which also means to break similar peers apart, teachers might be working against children’s tendency to form homophily in their friendship networks. The friendship literature suggests that similarity is what contributes to the sense of security and intimacy between friends (Newcomb and Bagwell, 1995). If similarity is the prerequisite for friendship building, it might take mixed-ability dyads longer to develop some level of similarity than same-ability dyads before they form friendships with each other. Same-ability dyads who were already friends might also have fewer opportunities to interact in the classroom due to the heterogeneous grouping practice, which might cause their friendship relationships to be weakened over time.

Consistent with the previous literature (Card et al., 2008; Sebanc et al., 2007; Underwood, 2007), boys showed a greater tendency than girls to engage in peer conflicts, whereas girls were likely to have more friends than boys. Moreover, a significant gender moderation effect was found in the relation between teachers’ heterogeneous ability grouping and children’s peer conflicts. Girls were found to engage in fewer peer conflicts if their teachers highly valued the heterogeneous ability grouping strategy, whereas boys’ experience with peer conflicts did not seem to be affected by this grouping strategy. Working with a diverse group of peers might require more advanced social skills (e.g., such as perspective taking, negotiation, or prosocial skills) than working with same-ability groups. Girls may already have possessed more social skills than boys (Van der Graaff et al., 2014; Jenkins and Nickerson, 2019) to avoid unconstructive conflicts with their peers.

It is surprising that maintaining existing friendships and forming new friendships did not show significant effects on changes in children’s friendships or peer conflicts. The null effects of these relationship-based grouping strategies counter against the physical proximity assumption (Homan, 1974) that children who are seated next to each other or work in the same group can know each other better, which then facilitate relationship building. One possible explanation is that teachers did not consider these grouping strategies important (see Table 2) and therefore did not utilize these strategies frequently enough to make an impact on children’s peer social experiences in the classroom. Alternatively, our finding might suggest that the link between physical proximity and relationship building may not be linear. The literature of seating charts supports this conjecture. It has shown that by placing children with a negative relationship in closer proximity for an extended period of time, even though rejected children became more liked by their peers (Van den Berg et al., 2012), the intervention classroom exhibited more aggression and less cooperation among classmates than their control counterparts (Braun et al., 2020). Future research should further examine other factors that may potentially alter the direction of influence of physical proximity, such as children’s characteristics, social climate, and different types of relationships.

It is important to note that the effects of teachers’ grouping strategies were examined by controlling for teachers’ self-efficacy for managing peer relationships. Ryan et al. (2015) showed that teachers with higher self-efficacy for creating a positive social climate, facilitating students’ friendship, and handling social problems were more likely to provide better instructional supports for students. Controlling for individual difference in managing peer relationships allows us to be more precise about identifying the social impacts of teachers’ grouping strategies.

### Limitations and Directions for Future Research

Despite the significance of the current study, we acknowledge several study limitations. First, teachers’ attitude toward grouping strategies might be in part contingent on the salience of child characteristics associated with those grouping strategies. For example, behavioral problems are highly noticeable than children’s friendship patterns, and many teachers have shown a poor understanding of their children’s friendship patterns in classrooms (Gest, 2006; Pearl et al., 2007). This may explain why teachers rated the separating behavioral problems strategy higher than the friendship building strategies. Qualitative or mixed methods approaches can be implemented in the future to further understand teacher beliefs of these grouping strategies.

Second, the current study measured grouping strategies based on teachers’ report instead of their actual grouping practices in the classroom. It is possible that even if teachers rated high on a grouping strategy, this rating may or may not be in alignment with their actual grouping practices. We chose to rely on teacher report in part because of the methodological challenge in observing teachers’ actual grouping practices in relation to their knowledge of children’s behavioral problems, ability level, and particularly existing relationships. However, future efforts in this area should continue to explore valid approaches to examining the connections between teachers’ attitude toward grouping strategies and their actual grouping practices.

Third, in this study we examined children’s friendship development based on the number of peer nominations that a child received. In this way, children’s friendship patterns were measured by perceptions from their classroom peers, which assured some level of reliability and objectivity. However, we acknowledge that other dimensions of friendship relationships can be equally important and deserve future inquiry, such as reciprocal vs. unilateral friendships and friendship quantity vs. quality. Finally, our findings on the gender moderation effect are largely exploratory without *a priori* theoretical hypotheses. Our main focus was to identify possible gender differences in the relationship between teachers’ grouping strategies and peer social experiences, which we anticipate will set the stage for future inquiry.

## Conclusion

This study documents changes in young children’s peer social experiences in early elementary classrooms, reveals how these changes are related to teachers’ grouping strategies, and explores whether these grouping strategies differentially mediate the social experiences of girls and boys. Since the pioneering research of teacher’s grouping strategies conducted by Gest and Rodkin (2011) in first, third, and fifth grade classrooms, the current study is the first endeavor to extend the literature on younger children’s peer social experiences (kindergarten to third grade), and is the first study that explores gender moderation of teacher influence. Overall, our findings show more differences than similarities with Gest and Rodkin’s pioneering work, which may indicate that teacher’s influence on children’s peer social experiences changes along the trajectory of children’s social development.

## Data Availability Statement

The raw data supporting the conclusions of this article will be made available by the authors upon request.

## Ethics Statement

The studies involving human participants were reviewed and approved by The Ohio State University. Written informed consent to participate in this study was provided by the participants’ legal guardian/next of kin.

## Author Contributions

SK and T-JL conceptualized the study. SK conducted data management, analyses, literature review, and writing. T-JL contributed to writing and guided SK on data analyses and literature review. JC and JL contributed to data management and statistical analysis. KP, LJ, and JC provided critical review of the manuscript. LJ, T-JL, and KP acquired the financial support for the project leading to this publication. All authors read and approved the submitted version of the manuscript.

## Conflict of Interest

The authors declare that the research was conducted in the absence of any commercial or financial relationships that could be construed as a potential conflict of interest.
